# Painful plantar heel spur treatment with Co-60 teletherapy: factors influencing treatment outcome

**DOI:** 10.1186/2193-1801-3-21

**Published:** 2014-01-10

**Authors:** Timur Koca, Ayşen Aydın, Duygu Sezen, Hamit Başaran, Sibel Karaca

**Affiliations:** Regional Training and Research Hospital Radiation Oncology Department, Caykara caddesi, Erzurum, 25200 Turkey

**Keywords:** Painful plantar heel spur, Cobalt-60 therapy, Böhler’s angle, Fat pillow thickness, Visual analogue scale

## Abstract

**Background:**

Painful plantar heel spur (PPHS) is a benign disorder with painful heels as a result of plantar calcaneal bone spur. Exact etiological factors are still unclear. Treatment typically consists of osteoarthritis tretment schedules and surgical techniques. External radiotherapy is another treatment option. This study is aimed to determine effectiveness and treatment outcomes of external radiotherapy in patients with PPHS.

**Methods:**

Sixty-two patients with PPHS were analysed for radiotherapy success and other possible prognostic factors. All patients were treated with Co-60 units from parallel opposed lateral portals, to a total dose of 8 Gy. Responses to radiotherapy was assessed by visual analogue scale (VAS) of pain. Follow-up completed in December 2012 with 28 months median duration (range 22 to 35 months). Age, sex, patient number, spur settlement site, prior treatments, time interval between diagnosis and radiotherapy, pain scores (before and after radiotherapy), plantar fat-pillow thickness (PFPT; thickness of the plantar fat pad) and Böhler’s angle estimations were analysed.

**Results:**

Study included 53 female and 9 male patients with median age 57 (range 43–70). Time interval between PPHS diagnosis and radiotherapy were median 33 months (range10-60). Radiotherapy response time interval were 6 months (range 3–10 months). Responses to radiotherapy were no response in 13 patients (21%), partial response in 13 patients (21%)- pain relief below 50% and complete response - no pain in 36 patients (58%) respectively. Median PFPT of patients were 3.5 cm (range 1.20–4.50 cm). Complete response rate was statistically significant in patients whom PFPT is greater than 3.5 cm. The Böhler’s angle range is about 20–40 deg. Complete response rates were higher in patients with degree of Böhler’s Angle 30 and below.

**Conclusions:**

Simplicity of treatment, lack of acute adverse effects and low cost, seem to make radiotherapy one of the safest, cheapest and also an effective treatment modality for PPHS.

## Background

Painful plantar heel spur (PPHS), is a common cause of heel pain in adults. Plantar calcaneal exostosis results in painful plantar fasciitis and bilateral involvement is more common in females. The term “heel spur” derives from calcaneal spur which was first described by Plettner in the beginning of the twentieth century (Plettner, 1900). While “plantar heel spur” reflects bone formation at the plantar insertion of the plantar fascia and muscles, “dorsal heel spur” is exostotic bone formation at the insertion of Achilles tendon. The latter type is less common and sometimes asymptomatic. Both can develop in the same individual (Muecke et al., 2007). Usually patients are older than 40 years but few sporadic pediatric cases have also been reported (Daniels and Morrell, 2012). The most common cause of plantar heel pain is reported to be plantar fasciitis and calcanei are the most common sites for bony spurs. Although age, genetics, weight and activity have been studied, etiological factors are still not clear (Muecke et al., 2007).

Treatment modalities used in osteoarthritis treatment are generally used in treating patients with PPHS. Orthopedic shoes, corticosteroid or anesthetic injections, non-steroidal anti-inflammatory drugs, extracorporeal shockwave treatment are the commonly used treatment modalities. There are also several surgical techniques used for PPHS (Micke and Seegenschmiedt, 2004).

Another treatment option for PPHS is external radiotherapy. Anti-inflammatory effects of radiotherapy are important for low doses. Possible mechanism of the anti-inflammatory effects of low-dose fractionated radiotherapy in such benign disorders is as follows, modulation of E-selectin mediated adhesion to endothelial cells, a decrease in leukocyte adhesion (Hildebrandt et al., 2002), reduction of nitric oxide synthase activity and reduction of oxidative burst in activated macrophages (Hildebrandt et al., 1998;Schaue et al., 2002). Antiproliferative and immunomodulatory effects are important for fraction doses > 2 Gy (Trott and Kamprad, 1999).

## Methods

Between February 2010 and December 2012, 62 patients with PPHS were analyzed for radiotherapy success and other possible prognostic factors. Patients were included into the study if meeting the following criteria: (1) Symptoms and clinical diagnosis of a painful heel spur; (2); duration of symptoms more than 6 months (3) radiologically proven heel spur; (4) Karnofsky performance status ≥ 70; (5) age ≥ 40 years. Patients who had previous radiotherapy or trauma to the foot and patients with severe psychiatric disorder, pregnancy and rheumatic and/or vascular diseases were excluded from the study. All of the patients were treated with Cobalt-60 units with once daily fractionation for two days, using parallel-opposed lateral portals, to a total dose of 8 Gy. The same target volume definition was used for all patients. Standard treatment volume included the whole calcaneus, plantar fascia insertion and the Achilles tendon insertion with appropriate fall off. Typical field sizes were ranged from 56 cm^2^ (7 × 8 cm) to 63.75 cm^2^ (7.5 × 8.5 cm). Patients were positioned in the frog leg position. Center of the treatment field was placed on the heel spur. Treatment field design was made using a conventional treatment simulator. Decreased pain free motions related to calcaneal heel spurs were indication for radiotherapeutic approach. None of the patients had received former radiotherapy for PPHS. All patients were informed about the side effects of treatment and possible carcinogenic risk of therapy. Written informed consents were signed by all patients before entering the trial.

Pain response to radiotherapy was assessed by VAS for pain, ranging from 0 to 10, in which zero means no pain and 10 means the worst imaginable pain.

Follow-up was completed in December 2012; the median duration of follow-up was 28 months (range 22 to 35 months). All patients were followed-up in the Radiotherapy Center of the Erzurum Regional Training & Research Hospital. Data were obtained by clinical examination, questionnaire and telephone interviews.

Age, sex, location of the spur, prior treatments, time interval between diagnosis and radiotherapy, pain scores (before and after radiotherapy), thickness of the heel pad and Böhler’s angle estimations were analyzed. The normal range of Böhler’s angle is about 20–40 degrees. It was formed by the intersection of 1) a line from the highest point of the posterior articular facet to the highest point of the superior tuberosity and 2) a line from the former to the highest point on the anterior articular facet.

All procedures followed were in accordance with the ethical standards of the responsible committee on human experimentation (institutional and national) and with the Helsinki Declaration of 1975, as revised in 2008 (5).

### Statistical analysis

Frequency tables were generated for categorical variables, and descriptive statistics (mean, standard deviation, median, minimum and maximum ranges) were calculated for numeric variables. Wilcoxon Signed Ranks Test were used to evaluate the significance of VAS score values before and after radiotherapeutic approach. Linear regression analyses were performed to determine most effective variable on VAS scores. All statistical analyses were performed using Statistical Package for Social Sciences, version 17.0 (SPSS Inc., Chicago, IL, USA). A p value lower than 0.05 was considered to be statistically significant.

## Results

The present study included 53 female and 9 male patients, with a median age of 57 years (range 43–70 years). Half of the patients had bilateral PPHS. Of the patients, 54 of them had received prior therapies. The treatment modalities used before radiotherapy were as follows, oral non-steroidal anti-inflammatory drugs (n = 14, 22.6%), corticosteroid injections (n = 8, 12.9%), physiotherapeutic interventions (n = 13, 21%), surgery (n = 6, 9.7%) and medication plus physiotherapeutic interventions (n = 13, 21%). The patients were referred to radiotherapy, primarily by orthopedic surgeons (n = 39; 63%), physical therapy and rehabilitation practitioners) (n = 13; 21%) and other physicians (n = 10; 16%), respectively.

The median time interval between PPHS diagnosis and radiotherapy was 33 months (range, 10–60 months). The time interval was especially long in patients living in the rural areas. Radiotherapy response time interval was 6 months (range 3–10 months). There was no response to radiotherapy in 13 patients (21%), partial response in 13 patients (21%) - pain relief below than 50% and complete response - no pain in 36 patients (58%). Baseline VAS score (before radiotherapy) and VAS score at 6 months follow-up were 7.40 ± 1.42 and 2.15 ± 3.00, respectively.

The median PFPT was 3.5 cm (range 1.20–4.50 cm). Complete response rate was significantly higher in patients with a PFPT > 3.5 cm, compared to that of patients with a PFPT ≤ 3.5 cm. A possible inverse relationship between radiotherapy response and Böhler’s angle was also examined. Complete response rates were higher in patients with a Böhlers angle ≤ 30 degrees.

Wilcoxon Signed Ranks Test is performed to evaluate probable significant difference between VAS scores before and after radiotherapy. Wilcoxon Signed Ranks Test results revealed; Z value: -6.175 and P: 0.000, evaluated as statistically significant. After linear regression analyses, R^2^ = 0.749, Regression Model; VAS score = −12.926 + 0.494 × Böhlers Angle. The result of variance analyses were; F = 178.749 and P = 0.000 and regression model were accepted as statistically significant. Linear regression analyses showed that the most effective variable on VAS changes were Böhler’s angle. Determination coefficient (R^2^) was 0.749 and revealed that 74.9% of the VAS score changes were resulting from difference in Böhler’s angle.

A very strong inverse relationship was present between pain radiation response and Böhler’s angle; a Böhler’s angle ≤ 30 degrees correlated with an increased response to radiation therapy. In addition, there was a strong positive relationship between the PFPT and response to radiotherapy; a PFPT > 3.5 cm correlated with increased radiation response.

Patient characteristics and the results of the multivariate analysis are summarized in Table 1 and Table 2, respectively.

**Table 1:** **Patient characteristics**

Patient characteristics	Mean ± SD or n (%)
**Age (years)**	57 ± 7,9
**Gender**	
Females	53 (85.5)
Males	9 (14.5)
**Location of the heel spur**	
Right	17 (27.4)
Left	16 (25.8)
Bilateral	29 (46.8)
**Prior therapies**	
Medical	22 (35.5)
Physical therapy	13 (21)
Surgery	6 (9.7)
Medical + physical therapy	13 (21)
**PFPT**	3.5 ± 0,8
**Böhler’s angle**	30 ± 5,2
**VAS scores**	
Before radiotherapy	7.40 ±1.42
After radiotherapy	2.15 ± 3

VAS: Visual Analogue Scale.

PFPT: Plantar fat pillow thickness.

Data are presented as mean ± standard deviation or n (%), where appropriate.

**Table 2:** **The results of the correlation analysis**

VAS	Böhler’s angle	PFPT
Correlation coefficient	0.865	- 0.635
Significance	0.000	0.000

## Discussion

Radiotherapeutic application in benign disorders is in use for near a hundred years in central Europe, and the patients with PPHS constitute an important part of the patients undergoing radiation therapy. However, radiotherapy is still the last preferred treatment approach for refractory cases especially in countries other than those in the central Europe (Micke and Seegenschmiedt, 2004; Miszczyk et al., 2007).

To date, possible carcinogenic risk of radiation therapy have been investigated in many trials, but, the risk was not as high as it was feared (Muecke et al., 2007; Surenkok et al., 2006). Radiotherapy fields used to treat plantar heel spurs are too small and the total doses are much lower than those used for malignant diseases.

A recent randomized trial published by Niewald et al. in (2012), comparing a standard dose with a very low dose, showed the clear superiority of the standard dose arm over the low dose arm, concerning pain relief as well as quality of life (Niewald et al., 2012). The superiority of the standard dose arm may be explained by the anti-proliferative and immune-modulatory effects of radiation gaining importance for doses > 2 Gy/fraction (Trott and Kamprad, 1999).

Numerous retrospective studies have shown that radiotherapy has a good analgesic effect in PPHS, but, radiotherapy was not standardized for time, fractionation, total radiation dose and portal design between patients in any of these trials (Muecke et al., 2007; Miszczyk et al., 2007; Niewald et al., 2012).

The calcaneus, which is the largest of the tarsal bones, articulates with the cuboid bone anteriorly and the talus bone superiorly. It transmits the majority of the body’s weight from the talus to the ground. Böhler’s angle may have a possible role in the development of heel spurs. This study is the first one that investigated the possible relationship between Böhler’s angle and the development of heel spurs. The importance of calcaneal loading, especially during walking and running, arises the importance of Böhler’s angle. However, trabecular architecture of the calcaneal bone also has a major importance. This architecture also predicts force transmission through the foot (Giddings et al., 2000).

Micke O et al., contradicted an old paradigm that orthovoltage with its higher bone and soft tissue absorption should be superior in outcome compared with linear accelerator photons (Micke and Seegenschmiedt, 2004). They predominately used linear accelerators with low energies between 4 and 9 MV in their patterns of care study. However, between economic disadvantages, non-diffuse distribution of the doses are also a disadvantage for linear accelerators when compared with Cobalt units.

## Conclusion

Simplicity of treatment, lack of acute and or long-term adverse effects and low cost, seems to make radiotherapy one of the safest, cheapest and effective treatment modality for PPHS. No acute and or long-term side effects were observed in this study.
